# Wordless intervention for epilepsy in learning disabilities (WIELD): study protocol for a randomized controlled feasibility trial

**DOI:** 10.1186/1745-6215-15-455

**Published:** 2014-11-20

**Authors:** Marie-Anne Durand, Bob Gates, Georgina Parkes, Asif Zia, Karin Friedli, Garry Barton, Howard Ring, Linda Oostendorp, David Wellsted

**Affiliations:** Centre for Lifespan and Chronic Illness Research, Department of Psychology, School of Life and Medical Sciences, University of Hertfordshire, College Lane Campus, Hatfield, AL10 9AB UK; The Dartmouth Institute for Health Policy and Clinical Practice, 37 Dewey Field Road, Hanover, NH 03755 USA; University of West London, Institute for Practice, Interdisciplinary Research and Enterprise (INSPIRE), A410, St Mary’s Road, Ealing, London, W5 5RF UK; Hertfordshire Partnership University NHS Foundation Trust, 99 Waverley Rd, St Albans, Hertfordshire AL3 5TL UK; Norwich Medical School and Norwich Clinical Trials Unit, Faculty of Medicine and Health Sciences, Chancellor’s Drive, University of East Anglia, Norwich, NR4 7TJ UK; Department of Psychiatry, University of Cambridge School of Clinical Medicine, Cambridge Biomedical Campus, Box 189, Cambridge, CB2 2QQ UK

**Keywords:** Epilepsy, Learning disability, Intervention, Feasibility trial, Clinical protocol

## Abstract

**Background:**

Epilepsy is the most common neurological problem that affects people with learning disabilities. The high seizure frequency, resistance to treatments, associated skills deficit and co-morbidities make the management of epilepsy particularly challenging for people with learning disabilities. The Books Beyond Words booklet for epilepsy uses images to help people with learning disabilities manage their condition and improve quality of life. Our aim is to conduct a randomized controlled feasibility trial exploring key methodological, design and acceptability issues, in order to subsequently undertake a large-scale randomized controlled trial of the Books Beyond Words booklet for epilepsy.

**Methods/Design:**

We will use a two-arm, single-centre randomized controlled feasibility design, over a 20-month period, across five epilepsy clinics in Hertfordshire, United Kingdom. We will recruit 40 eligible adults with learning disabilities and a confirmed diagnosis of epilepsy and will randomize them to use either the Books Beyond Words booklet plus usual care (intervention group) or to receive routine information and services (control group). We will collect quantitative data about the number of eligible participants, number of recruited participants, demographic data, discontinuation rates, variability of the primary outcome measure (quality of life: Epilepsy and Learning Disabilities Quality of Life scale), seizure severity, seizure control, intervention’s patterns of use, use of other epilepsy-related information, resource use and the EQ-5D-5L health questionnaire. We will also gather qualitative data about the feasibility and acceptability of the study procedures and the Books Beyond Words booklet. Ethical approval for this study was granted on 28 April 2014, by the Wales Research Ethics Committee 5. Recruitment began on 1 July 2014.

**Discussion:**

The outcomes of this feasibility study will be used to inform the design and methodology of a definitive study, adequately powered to determine the impact of the Books Beyond Words intervention to improve the management of epilepsy in people with learning disabilities.

**Trial registration:**

http://ISRCTN80067039 (Date of ISRCTN assignation: 23 April 2014).

## Background

### Rationale

People with learning disabilities experience a disproportionate burden of ill health and are affected by twice the number of health issues prevailing in the general population: higher incidence of long-term conditions and health risks, poorer health outcomes and an increased risk of premature death [1–4]. Epilepsy is one of many long-term conditions affecting people with learning disabilities that burdens their lives and hinders their quality of life [5]. It is considered the most common neurological disorder in people with learning disabilities, with a reported prevalence of 16 to 44% [2, 5–8], compared to 0.4% to 1% in the general population [9, 10].

The clinical management of epilepsy in people with learning disabilities is complex, due to both the clinical characteristics of the condition, as well as physical and cognitive impairments affecting them. Seizures are frequent, atypical and often severe. Approximately 70% of all seizures are refractory to treatment [11–14]. Seizures are less well controlled than in the general population of people with epilepsy, and may be accompanied by co-morbid health, mental health, sensory-motor and communication issues [15–17]. Poorly controlled epilepsy can severely affect social relationships, work, daily activities, quality of life and mortality [18–20]. As a result, young people and adults with learning disabilities and epilepsy face higher risks of premature and avoidable death related to epilepsy than the general population [21]. In addition, research shows that poorly controlled seizures and seizure frequency are associated with increased costs [22]. Pennington *et al.* recently demonstrated increased costs in people with epilepsy and learning disabilities [23], with a total estimated cost per patient, per annum, of £64,000 (including accommodation and basic care).

Managing long-term conditions is challenging for all patients, irrespective of their abilities and co-morbidities. However, it is even more complex for those with learning disabilities, who may not have the knowledge, skills and support required to assess potential risks and cope with recurring seizures, prescribed medications and regular doctor appointments. Research suggests that access to specialist services is poor for people with epilepsy and learning disabilities [24]. For carers and families who are not trained in supporting self-management, caring for adults with ‘dual disabilities’ can be extremely challenging and increase the caregiver burden [5]. Despite its high incidence, little is known about the management of epilepsy in people with learning disabilities, and those who support them. Research suggests that people with learning disabilities and epilepsy lack skills training adapted to their needs and intellectual abilities, and often fail to adequately manage and control their epilepsy [20]. The standard of care offered to people with learning disabilities and epilepsy is significantly poorer than in the general population, as is the case for most disease areas affecting people with learning disabilities [24, 25].

Guidelines from the National Institute for Health and Care Excellence (NICE) state that patients with epilepsy and learning disabilities: ‘should be empowered to manage their condition as well as possible, and should receive appropriate information and education about all aspects of epilepsy […] accessible to people with additional needs such as physical, sensory or learning disabilities’ [26]; p.9.

The Books Beyond Words booklet *Getting on with Epilepsy* has been designed to help people with learning disabilities and epilepsy understand and manage their illness through images that have been tested with people with mild to severe learning disabilities. The intervention is based on the assumption that people with learning disabilities who cannot read and have limited communication abilities are visually literate. Although the success and impact of the Books Beyond Words booklets (over 30 available) have been recognized by several awards and collaborations with established patient organizations, they have not as yet been formally evaluated in controlled settings or routinely adopted in the United Kingdom National Health Service (NHS). The lack of conclusive data on the effectiveness of educational interventions for people with epilepsy and learning disabilities warrants further investigation [24, 26].

### Aim

We aim to conduct a randomized controlled feasibility trial exploring key methodological, design and acceptability issues, in order to subsequently undertake a large-scale randomized controlled trial of the Books Beyond Words booklet as an intervention for epilepsy in adults with learning disabilities.

### Objectives

Our objectives are to:

1. Assess the feasibility of undertaking a randomized controlled trial of the Books Beyond Words intervention for epilepsy in people with learning disabilities.
2. To explore the feasibility of collecting resource use and quality of life data so as to inform the design of the health economics component of a future definitive trial.
3. Assess the study procedures’ and intervention’s acceptability among adults with learning disabilities, carers and health professionals.

## Methods/Design

### Study design and setting

We will use a two-arm, single-centre design and will conduct the study over a 20-month period at Hertfordshire Partnership University NHS Foundation Trust, United Kingdom. The trust runs five epilepsy clinics, managing 196 adults with learning disabilities and epilepsy. The patients are seen on average once a month. We will randomly allocate eligible participants to an intervention arm (provision of the Books Beyond Words booklet *Getting on with Epilepsy*) and control arm (routine information and services), and will recruit participants over six months.

### Participants

Eligible participants will be identified by the consultant psychiatrists who run the epilepsy clinics. Each psychiatrist will compile a log of potentially eligible patients from existing records and from new referrals using the following criteria.

#### Inclusion criteria

The inclusion criteria for this study are as follows:

1. Male and female patients, over 18 years of age.
2. A confirmed clinical diagnosis of epilepsy (according to medical notes) and at least one seizure over the past 12 months.
3. A confirmed clinical diagnosis of a learning disability (significantly below-average general intellectual functioning, and an IQ below or equal to 70).
4. Meaningful communication that enables the patient to tell or follow the Books Beyond Words story, as judged by the carer and/or health professional.
5. The carer is sufficiently proficient in English to read and complete the questionnaires with the patient.

#### Exclusion criteria

The exclusion criteria for this study are as follows:

1. Vision impairment.
2. Confirmed diagnosis of dementia.
3. Have used the Books Beyond Words booklet for epilepsy in the past 12 months.

## Consent

Each consultant psychiatrist at the participating epilepsy clinics will send an invitation letter and two information sheets to the carers of all eligible patients with learning disabilities and epilepsy registered at the clinics. In accordance with the Mental Capacity Act 2005 [27], an easy-read information sheet and invitation letter will be intended for the patient and another information sheet for the carer. In the information sheet, carers will be asked to encourage patients who have the capacity to read and understand (or to be read to and understand) the easy-read information sheet to do so. For patients who lack the mental capacity to understand the information sheet, consent will be obtained from the patient’s carer. It will be the carer’s role to use what they know of the patient’s wishes and feelings about research to decide whether they would like to take part in the study.

After a week, the research nurse will invite carers and patients by phone, to attend a meeting at the epilepsy clinic. During this appointment, the research nurse will review the patient information sheets with both the patient and the carer, and will take consent from the patient, with the carer’s involvement and advice, using an easy-read consent form. For patients who lack the capacity to consent, the research nurse will use a consultee declaration form, which will be completed and signed by the carer. The research nurse will also inform the patient’s GP of their participation in the feasibility trial.

### Randomization

During the initial appointment with the research nurse at the epilepsy clinic, and after consent has been taken, the carer will complete the baseline questionnaire. Randomization will be managed by the Norwich Clinical Trials Unit (NCTU). Once consent is obtained and the baseline data registered, the research nurse will allocate the patient to the intervention or control arm, as instructed by the randomization website. All authorized users, including the research nurse, will login using their unique username and password to a secure website to randomize each participant. The results will be stored in a study database on the Norwich Clinical Trials Unit (NCTU) secure server at the University of East Anglia (no user identifiable data will be stored in the randomization database). Web traffic will be encrypted using standard secure sockets layer technology. The randomization (1:1 allocation ratio) will be ‘blocked’ into groups of six codes to reduce the imbalance of control and intervention group participants at any time.

### Intervention group

**Figure 1:**
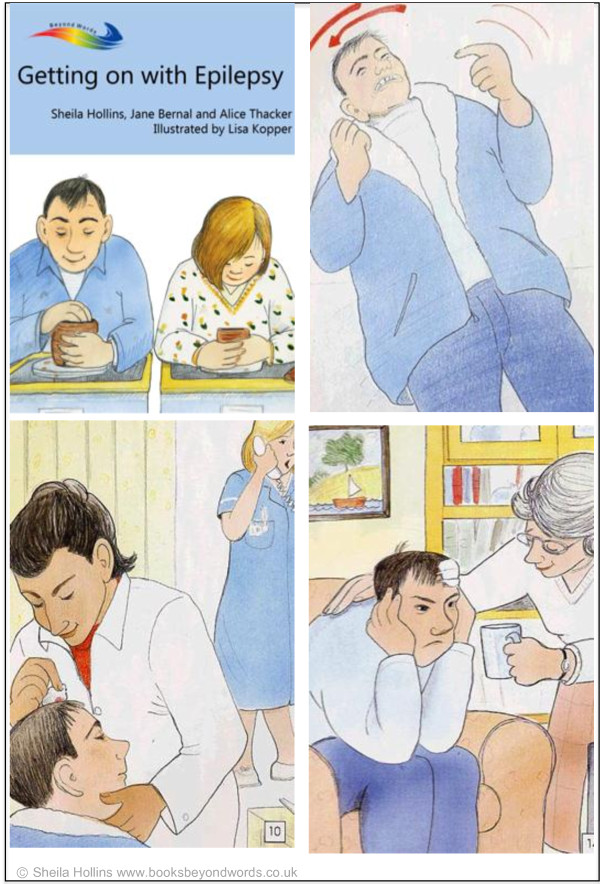
**The Books Beyond Words intervention.**

The Books Beyond Words booklet *Getting on with Epilepsy* uses pictures to tell the story of a young man with learning disabilities and epilepsy who progressively learns how to better manage his condition and recurrent seizures (see Figure 1). The intervention aims to improve seizure control, reduce the risk of falls and seizure-related injuries and improve quality of life. It also aims to illustrate best practice and promote access to relevant services. The Books Beyond Words booklet for epilepsy was developed over an 18-month period, by an editorial team of consultants in psychiatry and neurodevelopmental psychiatry, a speech and language therapist and an illustrator. They were supported by an advisory group of medical experts in the field of epilepsy and learning disabilities, by adults with learning disabilities and their carers, voluntary organizations and advocacy groups. Several iterations were developed and tested with a wide group of stakeholders, including adults with mild to severe learning disabilities and epilepsy [28].

#### How the Books Beyond Words intervention will be used

After giving consent and completing the baseline questionnaires, patients in the intervention condition will use the booklet with the research nurse and carer, in a quiet room at the epilepsy clinic. The nurse will go through the booklet with each patient at their own pace. Health professionals and carers can create their own story with the patient or use the story provided at the end of the booklet. The booklet contains instructions for use and a list of useful resources and services. The research nurse will show the first picture of the booklet to the person with learning disabilities, who should be encouraged to hold the book and turn the pages at their own pace. Depending on the patient’s communication abilities and reactions, it is advised that the research nurse will prompt them to say what is happening, whether this has happened to them before, and how they feel about it. We anticipate that it may take up to one hour to go through the booklet. If the intervention cannot be explored in one sitting, the research nurse will arrange a further appointment. The patient will also be given a copy to take home and explore with the carer and/or relatives.

It is recommended that the patient is given the opportunity to use the book at least twice more at home over the study duration. The research nurse will call each carer two weeks after randomization to remind them that the patient should be given opportunities to use the booklet at least twice more. Use of the book at home with the carer or other relatives will be monitored as part of the follow-up assessments. The carers will not receive any formal training in using the booklet, but will be able to watch an online video and training slides explaining how to use the book with the person they care for. A link to the training video and slides will be provided by the nurse at the end of the initial meeting. The research nurse will receive a half-day training course in using the intervention.

### Control group

Participants in the control group will receive routine information and services. The use of a picture book, such as the Books Beyond Words intervention or other educational package, will not be part of the routine consultation. After the study has terminated, patients in the control condition will be provided with a Books Beyond Words booklet to use with the epilepsy nurse specialist, the consultant and/or the carer.

### Outcome measures

**Figure 2:**
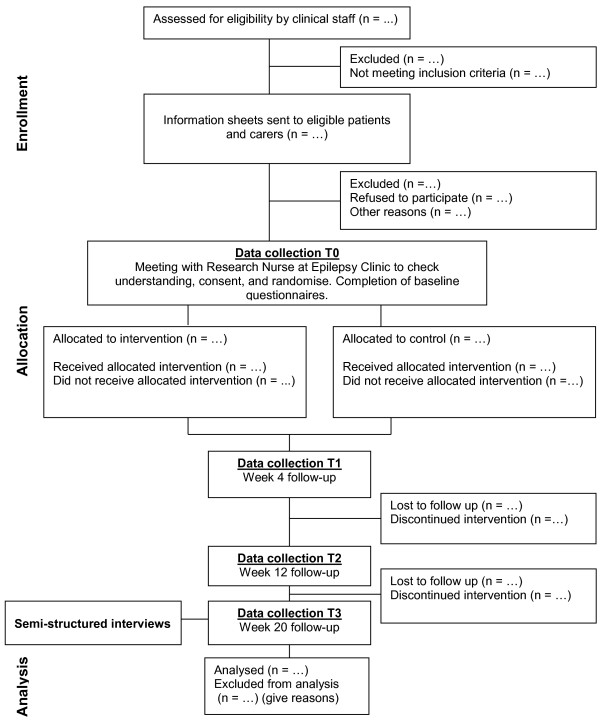
**Consolidated Standards of Reporting Trials (CONSORT) study flow-chart.**

Each patient will be assessed at baseline (T0), and at four (T1), 12 (T2) and 20 (T3) weeks after randomization (see Figure 2.). We will measure the following outcomes for both groups except where specified otherwise (see Table 1).

**Table 1:** **Study procedures according to study schedule**

Study procedures	Study period				
	Enrollment	Randomization	Post-randomization		
		T0	T1 week 4	T2 week 12	T3 week 20
**Eligibility screen**	X				
**Informed consent**	X				
**Allocation**		X			
**Intervention**		X			
**Rates of recruitment**		X	X	X	X
**Quality of life** (Epilepsy and Learning Disabilities Quality of Life, ELDQOL. scale)		X	X	X	X
**Demographic data**		X			
**Seizure severity** (using ELDQOL Seizure Severity Scale)		X			
**Seizure control** (diary cards)		X	X	X	X
**Feasibility and acceptability** (qualitative interviews)					X
**Resource use**		X			X
**Quality of life** (EQ-5D-5L scale)		X	X	X	X

#### Quantitative assessment

The quantitative assessment will examine the following:

1. Number of eligible patients identified and number recruited.
2. Variability of the primary outcome quality of life, using the Epilepsy and Learning Disabilities Quality of Life (ELDQOL) scale [29, 30]. The ELDQOL consists of four subscales: behaviour, seizure severity, mood and side effects.
3. Seizure control as a secondary outcome measure, assessed using a seizure diary.
4. Seizure severity as a secondary outcome measure, assessed using the seizure severity subscale of the ELDQOL scale.
5. Discontinuation rates across both arms and reasons.
6. Patterns of use of the Books Beyond Words booklet (for intervention group only).
7. Use of other epilepsy-related information.
8. Resource use and health-related quality of life using a context-specific resource use questionnaire and the EQ-5D-5 L scale [31].
9. Demographic data: age, sex, ethnicity, and living circumstances of the participant, information about the type of care provided by the carer and relationship to the participant.

#### Qualitative assessment

We will conduct semi-structured qualitative interviews at 20 weeks post-randomization, with a random sample of 15 patients and carers (or until data saturation), selected from both control and intervention groups, as well as all health professionals involved in the feasibility trial (n = 9). The semi-structured interviews will explore the feasibility and acceptability of the recruitment procedures (such as allocation, randomization and potential weaknesses of the design), use and perceived usefulness of existing resources and services, information and self-management support needs, perceived acceptability of the intervention and perceived barriers and facilitators to the use and dissemination of the intervention in routine care.

### Data collection and management

Data will be collected at baseline, four, 12 and 20 weeks post-randomization using a questionnaire and seizure diary completed by the carer, with the patient’s involvement whenever possible. The baseline questionnaire will be completed at the initial meeting with the research nurse. The follow-up questionnaires will be sent to the carer in the post and returned in a prepaid envelope. A research fellow will follow-up on patients and carers using the telephone at four, 12 and 20 weeks post-randomization to encourage the return of the questionnaires. A random sample of participants will also be invited to take part in an interview at 20 weeks post-randomization.

Data management will be provided by NRTU. The web-based data entry system will be built using Microsoft SQL Server tools. It will comply with the International Conference on Harmonization of Technical Requirements for Registration of Pharmaceuticals for Human Use Guideline for Good Clinical Practice (GCP), and will be restricted to authorized study team members and NRTU staff.

### Data analysis

#### Quantitative data

The analysis primarily aims to determine the feasibility of undertaking a full-scale randomized controlled trial and will seek to assess:

1. The number and demographics of eligible patients identified, screened, consented and randomized to each arm, and the relative performance in each clinic.
2. The demographics of patients who are excluded from the trial, reasons for exclusion and estimation of the proportion of eligible patients approached who agree to enter and complete the trial.
3. The proportion of completed study measures in each arm, measured from baseline to the last follow-up assessment.
4. The proportion of patients withdrawing from the study, measured from baseline to the last follow-up assessment.
5. The variation in the primary outcome measure that will be used, following Cocks and Torgerson [32], to determine whether a one-sided 80% confidence interval would exclude a 10% increase in the primary outcome (ELDQOL).
6. The frequency of use of the Books Beyond Words intervention, and other sources of information before and after randomization, and the extent to which use of the Books Beyond Words booklet influences outcome and other information resource use.

In parallel, a health economic assessment will be undertaken. We will monitor levels of resource-use and quality of life to inform the decision as to how costs and benefits should be measured as part of a future, more definitive study. NICE guidance recommends that costs are calculated from the perspective of the NHS and Personal Social Services (PSS) [33]. We will thereby seek to monitor levels of resource-use associated with the epilepsy booklet and the use of other NHS and PSS resource-items. Appropriate unit costs will subsequently be attached to all items of resource-use, to estimate the mean overall cost in each study-arm [34]. In line with NICE guidance, the EQ-5D [35] will be used to assess the effectiveness of each option. This will enable the quality-adjusted life year gain to be estimated. All analyses will be reported according to relevant CONSORT standards [36].

#### Qualitative data

The data collected in the semi-structured interviews will be analyzed using a two-step thematic content analysis derived from descriptive phenomenology [37, 38], assisted by the computer software NVivo.

### Sample size and feasibility of recruitment

The present study aims to assess the variation in the primary outcome measure between groups from baseline to the 12-week follow-up assessment, in order to determine whether the intervention is likely to achieve a 10% increase in the participants’ quality of life [32]. This would equate to an effect size of approximately 0.4, requiring a total sample size of 174 in a full study (1-β = 0.8, α = 0.05). Based on the requirement of 9% of the total sample size in each group, this indicates a required sample size of 16 per group, and will enable the exclusion of an effect size of <0. Given a potential 25% dropout rate, a sample size of 20 per group (40 participants in total) will be targeted. However, the study team, the selected study design and recruitment procedures will aim to minimize all potential dropouts whenever possible.

Recent audit data indicates that there were 196 patients with epilepsy and learning disabilities registered at the Hertfordshire Partnership University NHS Foundation Trust in May 2013. A preliminary screening exercise has already been undertaken by the lead consultant psychiatrist, at each epilepsy clinic, confirming that 112 out of 196 patients meet the study’s inclusion criteria. This does not include new referrals, who will be screened for eligibility once recruitment commences. The study will therefore aim to recruit approximately 36% of eligible patients.

### Confidentiality

This study will be conducted in accordance with the Data Protection Act 1998 [39] and the guidelines of the Declaration of Helsinki 1964 (updated Tokyo 2004) [40]. Access to the screening and randomization log, containing patient-identifiable data, will be restricted to authorized personnel and will be password-protected. Only GCP certified investigators approved by the Trial Steering Committee will be able to access the data. A unique patient identification number will be generated by the study web-based data entry system for each new patient entered in the system by the research nurse. This ensures anonymized data. A sequential number, starting at 001, will be generated for each new patient consented and entered into the study by the research nurse.

The original signed consent forms will be retained at the study site, in a locked cabinet. After all participants have been recruited, the researcher will collect baseline questionnaires from the epilepsy clinics. All case report forms and signed consent forms will be collected at the end of the study and stored in a locked cabinet at the University of Hertfordshire. Patient data relating to the study will be deleted or destroyed within three months of the end of the study. Anonymized study data will be archived by the University of Hertfordshire for five years after study completion, in line with standard research protocols. Investigators will not disclose patient data in any form to anyone not involved directly in the study. All electronic data will be stored on password-protected computers, and paper files will be stored in locked filing cabinets, both of which will be kept within electronically locked offices.

### Ethical considerations

This study includes people with learning disabilities who may or may not lack the capacity to consent. Whenever possible, consent will be obtained from the patient, with the carer’s involvement and advice, who will assess the participants’ capacity to take part, as required by the Mental Capacity Act. For patients who have the capacity to read or be read to and understand simple information (as determined by the carer and research nurse), an easy-read information sheet and invitation letter will be provided. These have been specifically designed according to the principles of the Mental Capacity Act (2005) [27], guidance from the Department of Health on producing easy-read information and guidance from the National Ethics Service on the design of study materials for adults without capacity. The information is paced, carefully structured, using simple language and images and with frequent references made to the carer. The easy-read consent form, invitation letter and information sheet have been reviewed by people from Hertfordshire Partnership University Foundation Trust, specializing in the development of easy-read information, and by members of our reference group, including people with learning disabilities, carers and members of the public. For patients who lack the capacity to consent, carers will be asked to complete and sign a consultee declaration form. Ethical approval for this study was granted on 28 April 2014, by the Wales Research Ethics Committee 5 (reference: 14/WA/0135).

## Discussion

This study is the first to assess the feasibility of undertaking a randomized controlled trial of an image-centric intervention, designed to facilitate the management of epilepsy in people with learning disabilities. The outputs of this feasibility study will be used to inform the design and methodology of a definitive study, adequately powered to determine the impact of the Books Beyond Words intervention to improve the management of epilepsy in people with learning disabilities. As advocated by NICE, The Royal College of General Practitioners and the Learning Disabilities Observatory, improving disease management and access to services of people with learning disabilities and epilepsy is essential and should become a priority in routine care. Although the feasibility study will not demonstrate whether or not Books Beyond Words interventions can achieve those goals, and provide value for money, this will be addressed by the definitive trial. The results will therefore have significant and long-lasting implications for clinical practice and service delivery, whereby Books Beyond Words interventions could become routinely embedded in the NHS, yielding significant benefits for patients, their carers and the wider population.

Further, the results of the feasibility trial will be of utmost importance to patient groups and clinicians in raising awareness of emerging research in learning disabilities and epilepsy, and demonstrating the potential acceptability of this intervention. Dissemination of the results will primarily be achieved through publication in peer-reviewed scientific journals, and reports intended for people with learning disability, their carers, families and the wider lay community, disseminated through national and local patient networks, including the Making it Better Group, Inclusion East, and the British Institute of Learning Disabilities (BILD). Promoting the involvement of people with learning disabilities in research and demonstrating potential benefits will be another focus of our dissemination strategy. We will work closely with the BILD and with the Patient Involvement in Research Group to raise awareness and increase the representation of patients with learning disabilities and their carers in research.

## Trial status

The trial set-up phase was completed at the end of May 2014. Recruitment started on 1 July 2014 and is expected to end in March 2015.

## Abbreviations

BILD: British Institute of Learning Disabilities
CONSORT: Consolidated Standards of Reporting Trials
CTU: Clinical Trials Unit
ELDQOL: Epilepsy and Learning Disabilities Quality of Life (scale)
GCP: Good Clinical Practice
ISRCTN: International Standard Randomized Controlled Trial Number
NHS: National Health Service
NICE: National Institute for health and Clinical Excellence
NCTU: Norwich Clinical Trials Unit
PSS: Personal Social Services
SQL: Structured Query Language
WIELD: Wordless Intervention for Epilepsy in Learning Disabilities.
